# Simple Technique of Exfoliation and Dispersion of Multilayer Graphene from Natural Graphite by Ozone-Assisted Sonication

**DOI:** 10.3390/nano7060125

**Published:** 2017-05-27

**Authors:** Zaw Lin, Paneer Selvam Karthik, Masaki Hada, Takeshi Nishikawa, Yasuhiko Hayashi

**Affiliations:** Graduate School of Natural Science and Technology, Okayama University, Okayama 700-8530, Japan; pspy2oke@s.okayama-u.ac.jp (Z.L.); pmsm2f0q@s.okayama-u.ac.jp (P.S.K.); hadamasaki@okayama-u.ac.jp (M.H.); nishikawa.takeshi@okayama-u.ac.jp (T.N.)

**Keywords:** multilayer graphene, ozone-assisted sonication, exfoliation, dispersion

## Abstract

Owing to its unique properties, graphene has attracted tremendous attention in many research fields. There is a great space to develop graphene synthesis techniques by an efficient and environmentally friendly approach. In this paper, we report a facile method to synthesize well-dispersed multilayer graphene (MLG) without using any chemical reagents or organic solvents. This was achieved by the ozone-assisted sonication of the natural graphite in a water medium. The frequency or number of ozone treatments plays an important role for the dispersion in the process. The possible mechanism of graphene exfoliation and the introduction of functional groups have been postulated. The experimental setup is unique for ozone treatment and enables the elimination of ozone off-gas. The heat generated by the dissipation of ultrasonic waves was used as it is, and no additional heat was supplied. The graphene dispersion was stable, and no evidence of aggregation was observed---even after several months. The characterization results show that well-dispersed MLG was successfully synthesized without any significant damage to the overall structure. The graphene obtained by this method has potential applications in composite materials, conductive coatings, energy storage, and electronic devices.

## 1. Introduction

Graphene, miracle material, is a two-dimensional carbon allotrope with a thickness of only one atom [1,2,3,4]. This material has attracted enormous attention in the scientific and applied research community due to its unique physical, mechanical, and chemical properties [5,6]. In the near future, graphene will be found as an indispensable material in many areas, such as composite materials, conductive films, supercapacitors, and nanofluids applications [7].

Several graphene synthesis methods have been developed since Novoselov and Geim discovered isolated graphene in 2004. The most common methods are liquid phase exfoliation, the reduction of graphene oxide, micromechanical exfoliation, chemical vapor deposition, and the exfoliation of graphite intercalation compounds in organic solvents [8,9,10,11,12]. Among these methods, the liquid phase exfoliation of graphite is the most promising way to produce graphene at a very low cost [13,14,15]. However, almost all reported methods have used surfactants, organic solvents, strong acids, and chemical reagents in order to exfoliate and stabilize in the specific medium [16,17,18]. Although the surfactants enhance the exfoliation and stability of the obtained graphene, there are some limitations to extensive applications, such as the toxicity and difficulty in removing the residual surfactants [19,20]. The cost and environmental impact of these reagents are the major challenges for these methods [21,22,23]. In addition, some degree of structural damage and the introduction of impurities from chemical reagents to the obtained graphene also limit its wide application [24,25]. The electrochemical exfoliation techniques for graphene and related materials have been reported to overcome the drawbacks of the conventional liquid phase exfoliation methods [26,27]. The quality and cost of the obtained graphene strongly depend on the synthesis method. Hence, it is important to choose the suitable method according to the desired applications. While most of the electronic applications use single- or few-layer graphene, the usage of multilayer graphene (MLG) is common in composite, conductive coating, and some other electronic applications. Very little research has been reported that relates the usage of more than 10 layers of graphene. Recently, the demonstration of the use of more than 30-layer MLG was reported [28,29]. Basically, the synthesis of MLG is much more cost effective and simple than single layer graphene. Depending on the applications, choosing the suitable graphene synthesis method is vital. 

Surfactant-free graphene synthesis in water is extremely difficult, and very little research has been done [30,31]. In the reported works, a kind of hydroxide solution or organic solvent was used to get optimum exfoliation conditions and stability. Consequently, it might introduce impurities and impact the properties of the obtained graphene. Recently, as a similar research, the direct exfoliation and dispersion of few-layer graphene in water using sonication method has been published. However, that method still has some challenges to realize in wide application owing to the long sonication time of 60 h [32]. In this current research, an ozone-assisted sonication method was proposed to synthesize MLG in water. The unique characteristics of this method are that it does not require the use of any chemical reagents, and it uses reasonable sonication time to synthesize well-dispersed MLG.

## 2. Materials and Methods 

### 2.1. Materials

Natural graphite powder (product code CNP-7, Ito Graphite Co., Ltd., Kuwano, Japan) with an average particle size of 6 μm and purity of over 99% was used as a starting material. Commercial grade oxygen was used as a gas source to produce ozone. Ultrapure water (Merck Millipore direct Q, Darmstadt, Germany) was used as a solvent in all steps. 

### 2.2. Experimental Setup for the Ozone Treatment

**Figure 1:**
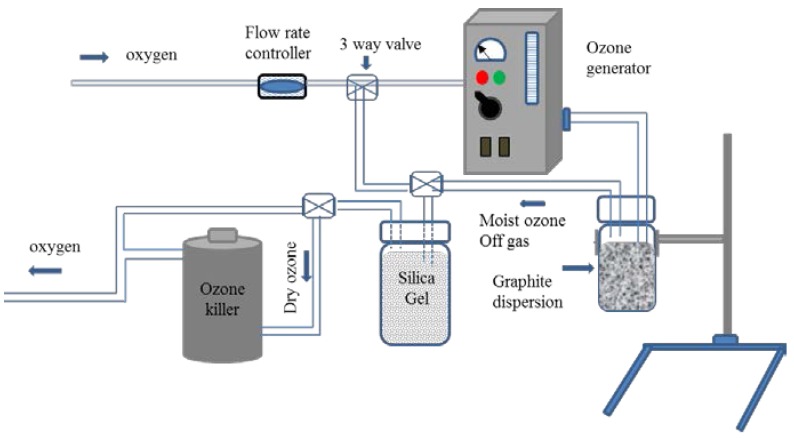
Experimental setup used for ozone generation and treatment of graphite suspension.

Figure 1 shows the experimental setup for ozone treatment of graphite suspension. The ozone generator (Ozone wave Syoken, Hiroshima, Japan) was used to produce ozone using commercial grade oxygen as a gas source. The oxygen flow rate regulator and the electric current of the ozone generator were set at 2 L/min and 0.2 A, respectively. The concentration of ozone in this condition was 1000 ppm, as measured by an ozone detector (AP-20, Kitagawa, Kawasaki, Japan). The ozone gas produced by the generator was directly fed into the Teflon vial containing the graphite suspension. The size of the Teflon vial was 45 mm in diameter and 65 mm in height, in which 100 mL of graphite suspension could be filled. The ozone killer containing manganese dioxide catalyst was used to convert ozone off-gas to oxygen. The moist ozone off-gas was dried by silica gel before entering ozone killer in order to avoid wetting to a catalyst, which can reduce the lifetime of the catalyst. Three-way valve installation was used to change the direction of gas flow when silica gel needed to be dried and refreshed.

### 2.3. Graphite Exfoliation by Sonication

**Figure 2:**
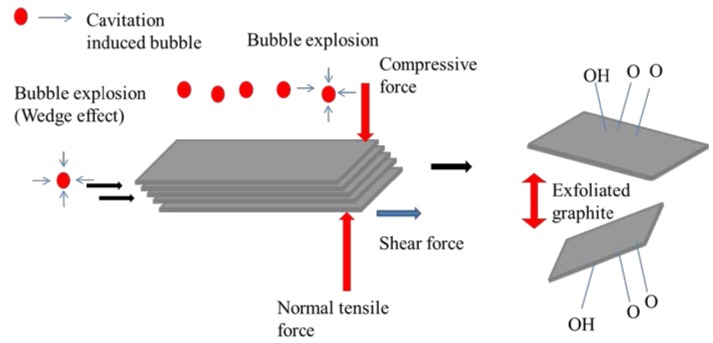
Illustration of the possible mechanism of graphite exfoliation.

An ultrasonic bath sonicator with frequency range 23 kHz and power of 80 watts was used to exfoliate natural graphite powders. The graphite powder (0.06 g) was added to 60 mL of ultrapure (millipore direct Q) water. During the sonication of graphite dispersion in water, the ultrasonic waves propagate through the graphite suspension and result in alternating high-pressure and low-pressure cycles. While the low-pressure cycle occurs, acoustic or ultrasonic waves create small vacuum bubbles in the suspension. Subsequently, these bubbles absorb the energy and collapse intensely during a high-pressure cycle. The effect of this cavitation phenomenon induces high-velocity micro liquid jets and shock waves which will generate normal and shear forces on the graphite [33]. Under the effect of intense forces and wedge action, graphite is expected to exfoliate as separated graphene layers. The schematic diagram of a possible mechanism for exfoliation [34] and dispersion of graphene by sonication is illustrated in Figure 2. In addition to this cavitation phenomenon caused by sonication, the explosion of dissolved ozone bubbles might enhance the exfoliation process. The primary purpose of ozone treatment is to get well-dispersed graphene as soon as it is exfoliated from bulk graphite. Ozone treatment was conducted intermittently for ten minutes in every 3 h sonication time. Sonication was performed from 1 to 15 h and the dispersibility of exfoliated graphene was observed constantly during the process. For a comparison, 15 h sonication without ozone treatment and 1 h continuous ozone treatment of graphite were also done. The temperature of the dispersion medium was increased up to 40 °C, owing to the heat generated by the dissipation of sonic wave. Consequently, it can be expected to enhance the exfoliation of graphene in water. 

### 2.4. Characterization

Scanning Electron Microscopy (SEM, JSM 6060 LA, JEOL, Tokyo, Japan) was used to characterize the morphology of the exfoliated samples. The number of graphene layers was determined by atomic force microscopy (AFM, Bruker, Multimode 8, Karlsruhe, Germany) in tapping mode. Raman spectroscopy analysis was also conducted to investigate the bond quality and confirm the number of graphene layers. The SiO_2_ (100 nm)/Si substrates were used for all of the above-mentioned characterizations. The substrates were cleaned with acetone in an ultrasonic bath and subsequently rinsed with isopropanol and blown dry using a nitrogen gun. X-ray photoelectron spectroscopy (XPS, JEOL JPS 9030, Tokyo, Japan) analysis was performed to observe the functional groups and oxygen content of the exfoliated samples. Dynamic light scattering and zeta potential measurements (Zetasizer Nano ZS, Malvern, UK) were used to measure the size distribution and the dispersibility of the exfoliated samples.

## 3. Results and Discussion

**Figure 3:**
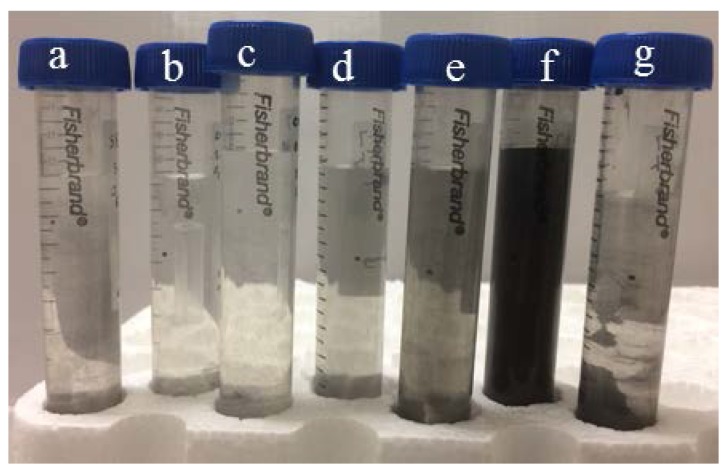
Photograph of centrifuge tubes showing exfoliated samples in different sonication and ozone treatment times: (**a**) 15 h sonication with 1 h continuous ozone treatment; (**b**) 3 h sonication with 2 × 10 min intermittent ozone treatment; (**c**) 6 h sonication with 3 × 10 min ozone treatment; (**d**) 9 h sonication with 4 × 10 min intermittent ozone treatment; (**e**) 12 h sonication with 5 × 10 min intermittent ozone treatment; (**f**) 15 h sonication with 6 × 10 min intermittent ozone treatment; (**g**) 15 h sonication without ozone treatment.

Figure 3 shows the photograph of centrifuge tubes showing the exfoliated samples in different sonication and ozone treatment times. The picture was taken after centrifugation of the exfoliated samples at 3000 RPM for 20 min. It can be seen that exfoliated samples could not disperse at 15 h sonication with 1 h continuous ozone treatment (Figure 3a), 3 h sonication with intermittent ozone treatment of 2 × 10 min (Figure 3b), 6 h sonication with intermittent ozone treatment of 3 × 10 min (Figure 3c), 9 h sonication with intermittent ozone treatment of 4 × 10 min (Figure 3d), and 15 h sonication without ozone treatment (Figure 3g) respectively. In contrast, slight dispersion can be observed at 12 h sonication with intermittent ozone treatment of 5 × 10 min (Figure 3e) and the sample was well-dispersed at 15 h sonication with intermittent ozone treatment of 6 × 10 min (Figure 3f). The well-dispersed sample was stable for several months without any significant agglomeration. The upper half of the well-dispersed sample that was subjected to 15 h sonication with intermittent ozone treatment of 6 × 10 min was taken for further characterizations. A relatively high concentration (0.12 mg/mL) of dispersed MLG was achieved. 

**Figure 4:**
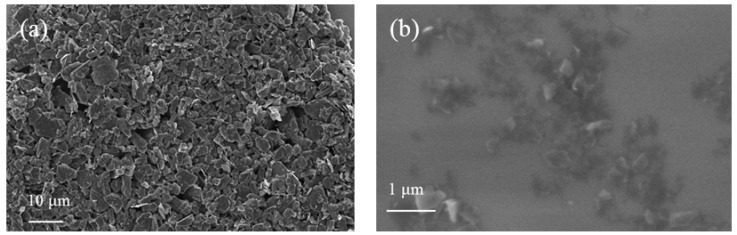
SEM images of (**a**) pristine graphite; (**b**) exfoliated graphene samples.

The morphologies and sizes of pristine and exfoliated graphite observed by SEM are shown in Figure 4. The samples were prepared by drop casting onto the substrate and dried at 80 °C for 30 min on a hot plate. It can be seen in Figure 4a that the majority of pristine graphite’s size was around 6 μm. The high contrast image could be taken at 5 kV acceleration voltage. Compared to pristine graphite, the image contrast of exfoliated graphene (Figure 4b) was weak to distinguish from the substrate at the same accelerating voltage. This is one of the proofs of achieving graphene. The majority of the graphene appeared dark pale that corresponds to MLG. In addition, some of the brighter ones that correspond to few-layer graphene were also observed [35].

**Figure 5:**
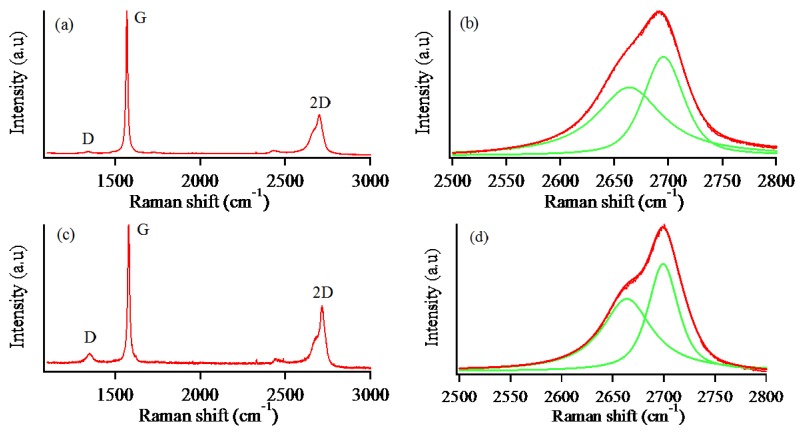
Raman spectra of (**a**) natural graphite; (**b**) deconvoluted 2D peak of natural graphite; (**c**) exfoliated graphene; (**d**) deconvoluted 2D peak of exfoliated graphene.

The Raman spectra of pristine graphite and exfoliated MLG are shown in Figure 5. Graphene can be identified in terms of bond quality and the number of layers by Raman spectroscopy analysis. It is one of the most reliable and non-destructive tools to characterize graphene. As can be seen in Figure 5a, there are three major Raman features of pristine natural graphite: D, G, and 2D bands which appear around 1322, 1567, and 2700 cm^−1^. The deconvolution of the 2D band in graphite shows two sub-peaks at 2655 and 2700 cm^−1^, as shown in Figure 5b. The spectrum of exfoliated graphene (Figure 5c) mainly exhibits three characteristic peaks—D, G, and 2D bands around 1348, 1577, and 2712 cm^−1^. Likewise, two similar sub-peaks appear at 2655 and 2700 cm^−1^ in the deconvoluted spectra of MLG, as seen in Figure 5d. The D and G bands are caused by disorder and the order or purity of graphite structure. The 2D band is the second order of the two-phonon process and can be used to determine the number of graphene layers. The value of *I*_2D_/*I*_G_ intensity ratio is very common and helpful to determine the number of graphene layers. The intensity ratio, *I*_2D_/*I*_G_, for single-layer graphene is around 2, bi-layer graphene is around 1, and multilayer graphene is less than 1. Pristine natural graphite exhibits *I*_2D_/*I*_G_ ratio of 0.31 with very low D band peak. Exfoliated graphene reveals higher *I*_2D_/*I*_G_ ratio (0.42) with a little higher D band peak compared to graphite. Based on the described intensity ratio, it can be determined that the exfoliated graphene is multilayer graphene with well-maintained graphitic structure. However, another confirmation like AFM measurement is necessary for clear understanding of the number of graphene layers.

**Figure 6:**
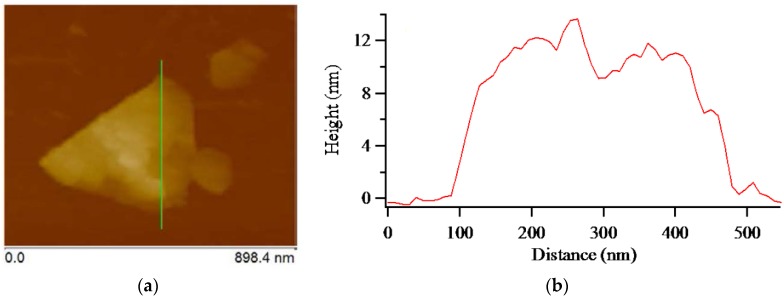
(**a**) Atomic force microscopy (AFM) image of the multilayer graphene (MLG); (**b**) Corresponding height profile of the MLG.

Figure 6 shows the AFM image of exfoliated graphene and its corresponding height profile. AFM measurement is the most suitable technique to investigate the number of layers and morphology of graphene. The height profile image can be used to get information about the thickness of the graphene layer and underlying substrate. The exfoliated graphene sample was deposited on the substrate by a dipping method and dried at 80 °C for 30 min on the hot plate. As can be seen in Figure 6a, b, approximately 13 nm thick and a few hundred nanometers wide MLG and some smaller and thinner graphene sheets can be observed. In the ideal natural graphite structure, carbon atoms are arranged in hexagonal rings stacked in an ABAB sequence. The inter-planer spacing of graphite (0.335 nm) is used as a reference value to estimate the number of graphene layers [36]. Depending on the sample preparation, substrate–graphene, and AFM probe–graphene interaction, some extent of variation might occur in the estimation of graphene layer through the thickness. In addition, the water adlayers that might increase the actual thickness of graphene might also be formed between substrate and MLG owing to the usage of water. Considering these variations, it was possible to estimate that the number of layers of the obtained MLG was around 26 to 38 layers. 

**Figure 7:**
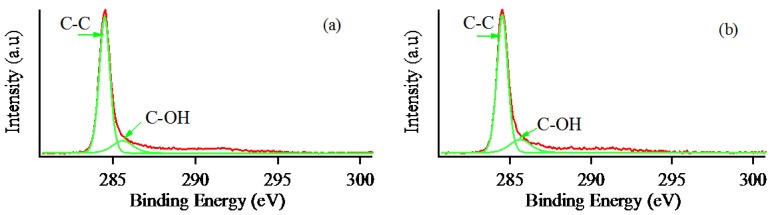
The X-ray photoelectron spectroscopy (XPS) spectra of (**a**) pristine graphite; and (**b**) multilayer graphene.

The XPS measurement was performed to understand the quantitative elemental, chemical state, and functional groups. The detailed C 1s analysis of pristine graphite and MLG are shown in Figure 7a,b. The C 1s region was deconvoluted into two components at 284.8 and 285.9 eV, corresponding to C–C and C–OH hydroxyl group. There are no obvious differences in the C 1s spectra of pristine graphite and the MLG obtained by this method. As shown in Table 1, the oxygen content was increased by nearly two times after exfoliation owing to the surface modification by ozone. This result implies a high-purity nature of the obtained MLG through the clear carbon signature without any severe oxidation. The oxygen content of dispersed MLG might be more than 4.48 atomic weight percent in the solution state. The samples for XPS measurement were dried on the hot plate for 30 min at 80 °C in the sample preparation step. In addition, XPS measurement has to be performed under very high vacuum. Consequently, the oxygen-containing functional group or oxygen content measured by XPS might be lower than those in the solution state. 

**Figure 8:**
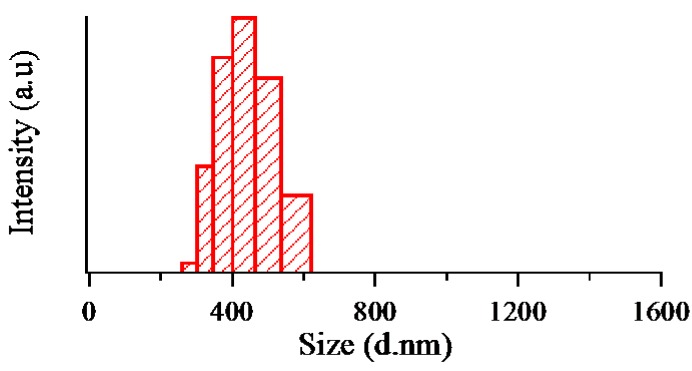
Size distribution of the dispersed multilayer graphene.

The size distributions of the obtained MLG were measured with a Zetasizer Nano Series particle size analyzer by the dynamic light scattering method. The disposable cuvette container (DTS 0012) filled with 3 mL of dispersed MLG was used for the measurement. As shown in Figure 8, the mean size of the MLG was found to be around 400 nm.

**Figure 9:**
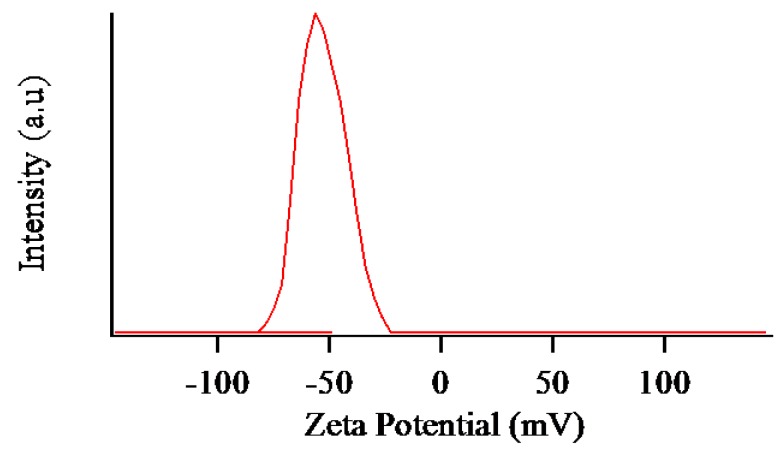
Zeta potential distribution graph of the MLG.

The stability of the dispersed MLG was measured by a zeta potential analyzer (the same device as for the DLS measurement). Disposable folded capillary cells (DTS 1070) filled with 2 mL of dispersed MLG were used for measurement. The zeta potential (ZP) is the key indicator to describe the stability of dispersed MLG and is caused by the net electrical charge in the medium. The magnitude of ZP describes the degree of electrostatic repulsion between the charged particles in a dispersed solution. Generally, a ZP value less than 30 mV (negative or positive) shows instability behavior of a dispersed solution and the occurrence of aggregation owing to the higher attractive force than repulsive force. In contrast, a ZP value higher than 30 mV indicates good stability, and will resist aggregation or flocculation. Figure 9 shows the ZP value of the obtained MLG dispersed in water. The result revealed that the dispersed graphene is highly negatively charged (−56.4 mV), apparently as a result of the decomposition of the ozone and water molecules. The obtained aqueous dispersion of the MLG showed good stability: There was no sign of the MLG agglomeration, even after more than five months. 

## 4. Conclusions

This study revealed a facile method to synthesize well-dispersed MLG without using any chemical reagents or organic solvents. The obtained MLG was found to be 13 nm thick and a few hundred nanometers wide with negligible structural damage. The dispersed MLG was stable, and no evidence of aggregation was observed, even after several months. These results were achieved using sonication-assisted ozone treatment. The number of ozone treatment times (frequency) is the key to achieving good dispersion. It can also be concluded that a minimum of 15 h sonication with intermittent ozone treatment of 6 × 10 min is required to synthesize well-dispersed MLG. The experimental setup was unique for this kind of ozone treatment—not only of graphene, but also many varieties of materials. This process does not produce ozone off-gas or any waste products to the environment. The MLG herein obtained can be used in composite materials, conductive films, nanofluids, and electronic applications.

## Figures and Tables

**Table 1:** Oxygen concentration of pristine graphite and MLG measured by XPS.

Sample	Concentration (Atomic Weight %)	
	Carbon	Oxygen
Pristine natural graphite	97.23	2.77
Multilayer graphene	95.52	4.48
